# De-Epithelization of the Human Amniotic Membrane Using a System Involving Ozonated Water and Ultrasound

**DOI:** 10.3390/bioengineering11100987

**Published:** 2024-09-29

**Authors:** Francisco Dimitre Rodrigo Pereira Santos, Bianca Akemi Kawata, Tatiana Regina de Oliveira Heinzelmann, Marcia Guelma Santos Belfort, Maycon Crispim de Oliveira Carvalho, Sílvia Móbille Awoyama, João Gomes de Oliveira Neto, Carlos José de Lima, Adriana Barrinha Fernandes

**Affiliations:** 1 Postgraduate Program in Biomedical Engineering, Biomedical Engineering Institute, Universidade Anhembi Morumbi (UAM), São José dos Campos 12247-004, São Paulo, Brazil; tatiheinzelmann@gmail.com (T.R.d.O.H.); marciguelma@hotmail.com (M.G.S.B.); mayconcarvalho20@gmail.com (M.C.d.O.C.); cdcfdlima@gmail.com (C.J.d.L.); fernandesabm@gmail.com (A.B.F.); 2 Department of Medicine, Universidade Estadual do Tocantins (UNITINS), Augustinópolis 77960-000, Tocantins, Brazil; 3 Instituto de Ensino Superior do Sul do Maranhão (IESMA), Imperatriz 65907-070, Maranhão, Brazil; 4 Coordination of Research and Technological Development from Instituto Nacional de Pesquisas Espaciais (COPDT/INPE), São José dos Campos 12201-970, São Paulo, Brazil; kawata.bianca@gmail.com; 5 Centro de Inovação, Tecnologia e Educação (CITÉ), São José dos Campos 12247-004, São Paulo, Brazil; silviamobille@gmail.com; 6 College of Pharmacy, Centro Universitário FUNVIC-UNIFUNVIC, Pindamonhangaba 12412-825, São Paulo, Brazil; 7 Postgraduate Program in Materials Science, Universidade Federal do Maranhã (UFMA), Imperatriz 65915-060, Maranhão, Brazil; joaogomes.quimico@gmail.com

**Keywords:** amnion, human amniotic membrane, de-epithelization, ozone, ultrasound, biomaterials, tissue engineering

## Abstract

The aim of this study was to evaluate whether a system involving ozonated water and ultrasound causes de-epithelization of the human amniotic membrane (HAM). The experiment protocol was carried out in four stages. Stage I was carried out to determine the duration of the experiment. Stage II comprised the first experiment, involving four groups of samples studied in triplicate: control/natural (IN), processed with ultrasound in a liquid medium (US), processed with ozonated water (O3), and processed with ozonated water combined with ultrasound (US_O3). Stage III was performed to confirm the results, following the same steps present in Stage II. Stage IV involved the use of oxygen to confirm the hypothesis. Histological analysis was carried out to verify whether the effects of O_2_ were similar to those of O_3_. The system was activated, and ozonation was carried out for 10 min, as in the previous experiment, reaching a concentration level of 3.0 mg/L. The samples were submerged and positioned in the reservoir and processed separately for 55 min. The biochemical properties were assessed using Fourier transform infrared spectroscopy, and the morphology was examined using histology and scanning electron microscopy. The spectra of the samples exhibited similarities; however, subtle changes were highlighted, such as smooth band shifts and intensity changes. The morphology indicated that ultrasound achieved more efficient HAM de-epithelialization compared to ultrasound combined with ozonated water and ozonated water alone. One plausible hypothesis for this observation is that cavitation represents the primary mechanism responsible for de-epithelialization. When ultrasound is combined with ozone, the bubbles generated by ozone gas reduce the cavitation effect. This study is pioneering as it demonstrates an ultrasound system capable of the efficient de-epithelialization of the HAM.

## 1. Introduction

The human amniotic membrane (HAM) is a thin film that is the innermost of the fetal membranes and is usually discarded after childbirth [1]. This material is formed of three histological layers: the epithelial layer, the basement membrane, and the avascular mesenchymal tissue composed of fibroblasts and collagen, which is also known as the stroma [2].

The HAM is composed of fibronectin, proteoglycans, glycosaminoglycans, laminins, and collagen types I, III, IV, V, VI, and VII, as well as fibroblasts and pluripotent stem cells, which aid in tissue growth and repair [3]. The participation of the HAM in the immunomodulation of inflammation has been related to the presence of interleukins (IL-4, IL-6, IL-8, and IL-10) and growth factors [4].

In recent years, clinical applications of the HAM have been widely studied in the field of tissue engineering due to its morphological characterization, biochemical properties, and therapeutic responses, which have attracted the interest of scientists and biomaterial engineers [5]. This membrane is already widely used in healing and pain-reducing processes for wounds around the world [6]. In a recent study, Fitriani et al. [7] reported on its use in the development of biomaterials for the correction of dermal alterations, the formation of living tissue for drug administration, the treatment of epidermolysis bullosa, the reconstruction of the urethra, regeneration in cases of calciphylaxis, and tendon healing. Additionally, there is promising evidence of its potential use in ophthalmology, cardiac and liver surgeries [8], bone regeneration [9], and cancer therapy [10].

For clinical use, the HAM needs to be properly processed with suitable disinfection/sterilization techniques to remove possible pathogens [11]. These processes often break peptide chains, compromising the integrity and function of the membrane [12]. Subsequently, these materials must be properly preserved. The most common techniques used for preservation are cryopreservation, dry freezing, and air drying. For some applications, de-epithelization is necessary the membrane without epithelium, promote increased rates of cell proliferation and differentiation [11,13].

HAM de-epithelization can be carried out using proteolytic enzymes, such as trypsin and dispase [14], which break the peptide bonds of proteins [15], promoting the complete removal of the epithelium and basement membrane [14,15]. Another widely used method in this process is the use of ethylenediaminetetraacetic acid (EDTA), which leaves areas of the epithelium intact and partially destroys the basement membrane [14]. Additionally, detergent-free protocols or the use of nonionic detergents, such as Triton X-100, can be effective in terms of HAM de-epithelization [16,17]. In a recently published study, Awoyama et al. [18] conducted research using a system involving ozonated water to disinfect HAM. Their data revealed that a 15 min ozonization period was sufficient to cause small morphological changes in the epithelial layer without causing significant alterations to the scaffold, making it a promising method for the de-epithelization of HAM [18].

Ozone gas (O_3_) is an allotrope form of oxygen (O_2_) and has great potential for use as an oxidizing agent. When this gas is dissolved in water, the bonds of O_3_ molecules are broken [19], generating reactive oxygen species (ROS) [20] that react with organic and inorganic compounds [21,22]. This mechanism increases the permeability of the cytoplasmic membrane that interacts with the cytoplasm, leading to cellular dissolution [23], like the interaction of gaseous O_3_ with epithelial tissue [24]. An important fact that favors the interaction of O_3_ with the HAM is that oxidation processes do not break the bonds between the amide groups of proteins [25].

Like O_3_, ultrasound is used in the processing of biological materials due to the generation of hydrogen peroxide (H_2_O_2_) and ROS, such as HO and HO_2_, which are involved in the degradation and inactivation of microorganisms [26]. These effects occur due to the mechanism of ultrasound in water. An alternating current under cyclic frequency conditions acting on a piezoelectric crystal generates mechanical waves [27]. Cavitation occurs through the propagation of sound waves [28], which have the functional capacity to act on tissues, resulting in increased cell membrane permeability [29], especially in endothelial cells [30]. In contrast, low-frequency ultrasound (24–40 kHz) enables the removal of blood elements and HAM de-epithelization [31].

Motivated by the small morphological changes that the use of ozonated water causes in the epithelial layer observed by Awoyama et al. [18] and the potential of ultrasound in terms of HAM de-epithelization shown by Milyudin et al. [31], this study used the system developed by Heinzelmann et al. [32], wherein the techniques can be used separately or combined. 

The aim of this study was to evaluate whether the system with ozonated water and ultrasound causes de-epithelization of the human amniotic membrane (HAM). The biochemical properties were evaluated by means of Fourier transform infrared spectroscopy (FT-IR) and the morphology was evaluated by means of histology and high-resolution field emission gun (FEG) scanning electron microscopy (SEM).

## 2. Materials and Methods

### 2.1. Study Type and Ethical Aspects

This research refers to an in vitro and comparative experimental study that encompasses a broader project approved by the Ethics Committee for Research of Universidade Anhembi Morumbi with the following approval number: 3.984.423 CAAE: 289370020.0.0000.5492. This study followed all determinations of Resolution n.190 of 19 July 2003 of the Agência Nacional de Vigilância Sanitária, which determines the technical standards for the operation of umbilical cord and placental blood banks [33].

### 2.2. Sample Collection

**Figure 1:**
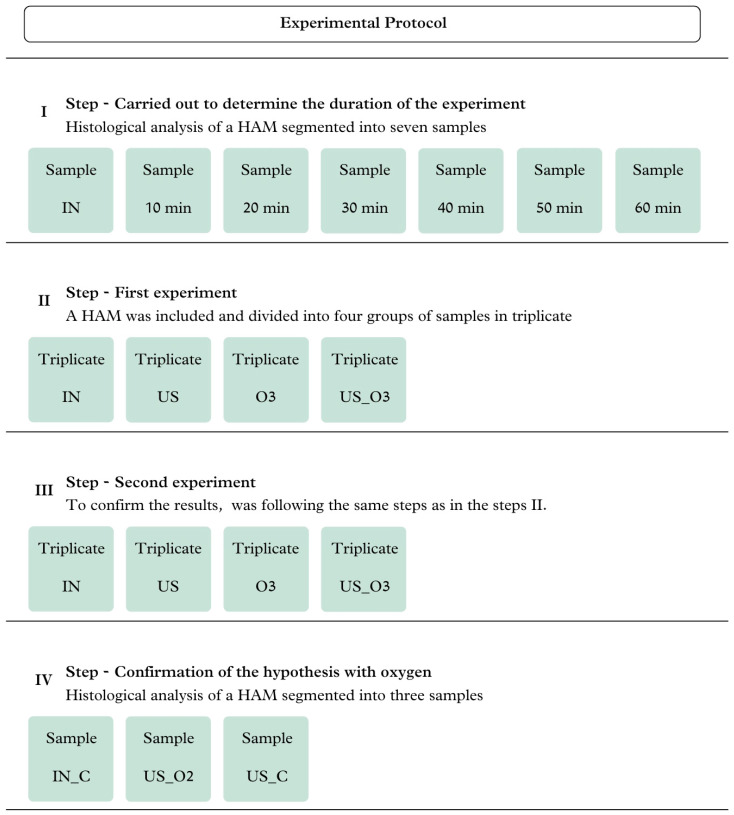
Experiment protocol steps.

The collection was carried out at Santa Casa de Misericórdia de Pindamonhangaba, São Paulo, Brazil. Four HAMs were collected, one for each phase of the study (Figure 1). The HAMs were fresh and aseptically obtained from cesarean sections of seronegative donors (for syphilis, hepatitis B, toxoplasmosis, and HIV) aged between 28 and 30 years. The donations were made voluntarily for the research, after signing an informed consent form.

The material was stored in a glass vial containing saline solution (NaCl 0.9%), and was subsequently stored in a thermal container at a temperature between 10 and 15 °C and transported to the Disinfection and Sterilization Laboratory of the Center for Innovation Technology and Education (CITÉ) in São José dos Campos, São Paulo, Brazil. The membranes were manually washed in NaCl 0.9% solution 10 times to remove possible biological residues.

### 2.3. Experimental Procedure

The experimental protocol was carried out across four stages, as described in Figure 1. Stage I was carried out to determine the duration of the experiment. For this, seven samples were segmented, including one control sample and six study samples, which were submitted to the system involving ozonated water combined with ultrasound for different lengths of time (10, 20, 30, 40, 50, and 60 min). Stage II comprised the first experiment. Here, an HAM was divided into four groups of samples that were assessed in triplicate: control/natural (IN), processed with ultrasound in liquid medium (US), processed with ozonated water (O3), and processed with ozonated water combined with ultrasound (US_O3). Stage III was performed to confirm the results; for this, an HAM was used, following the same steps as used in Stage II. Stage IV was carried out using oxygen to confirm the hypothesis. Histological analysis was carried out to verify whether the effects of O_2_ were similar to those of O_3_; in this stage, an HAM was segmented into three samples: one control/natural (IN_C), processed with O_2_ and ultrasound (O2_US), and processed with ultrasound in a liquid medium (US_C).

The same anatomical region of the membrane was used for all experiments, as recommended in the study developed by Weidinger et al. [34]. The HAM samples were cut using sterile scalpel blades into pieces with a width of 2 cm and a length of 6 cm, totaling an area of 12 cm^2^. The samples were fixed on a support made from polyvinyl chloride (PVC).

The same equipment was used as that described in the study conducted by Heinzelmann et al. [32]. This consisted of a system involving ozonated water and ultrasound. The ozone generator (model MS3G, Medical Systems Ltda, São José dos Campos, Brazil) was connected to an O_2_ cylinder with a flow rate adjusted to 0.25 L/min, resulting in a concentration of 48 mg/L. O_3_ was dissolved in the distilled water present in the system reservoir using a Venturi injector integrated into the equipment. An ultrasound generator head with a piezoelectric crystal (40 kHz 50 W acoustics) was attached to the base of the main reservoir.

Inside the exhaust hood, the system reservoir was filled with 700 mL of distilled water at a temperature between 17 and 20 °C. For the O3 and US_O3 samples, the system was activated, and ozonation was performed for 10 min, which reached a concentration level of 3.0 mg/L before the start of the experiment. The three samples were submerged and positioned in the center of the reservoir and the groups were processed separately for 55 min.

The system operated with an average water speed rate in the main reservoir of 2 cm/s. The ozone production rate was 12 mg/min; considering the production rate and the 55 min ozone exposure time, the mass of O_3_ delivered was 660 mg. Considering the HAM area of 12 cm^2^, the O_3_ dosage applied per cm^2^ was 55 mg/cm^2^.

### 2.4. Analysis

#### 2.4.1. FT-IR Analysis

FT-IR spectra were obtained using a PerkinElmer FT-IR/NIR Spectrometer Frontier in the spectral range of 400 to 4000 cm^−1^ with 45 scans and a spectral resolution of ±4 cm^−1^ using a universal attenuated total reflection (UATR) accessory. The spectra were obtained in quintuplicate, and the median was determined for subsequent analysis at the Instituto de Estudos Avançados do Mar (IEAMar) of Universidade Estadual Paulista (Unesp) in São José dos Campos, São Paulo, Brazil.

#### 2.4.2. Histological Analysis

For histological analysis, the samples were fixed with 10% formalin solution of the brand Dinâmica/Química Industrial^®^ and then dehydrated, clarified, impregnated, and embedded in paraffin blocks. The second part of the material preparation was sectioned using a rotary microtome and stained with hematoxylin and eosin. For slide visualization, an optical microscope (Opticam^®^ Microscopy Technology, São Paulo, Brazil) was used; the images were obtained using OPTHD^®^ 1.0 software.

#### 2.4.3. SEM-FEG Analysis

After processing, the samples were fixed in a solution of the brand Dinâmica/Química Industrial^®^, with 2.5% glutaraldehyde in 0.2 M phosphate buffer and distilled water. The samples were then kept at a temperature between 2 and 8 °C for 48 hours until analysis. After this period, each sample underwent three washes of 30 min each using a solution with a 1:1 ratio of disodium phosphate, monosodium phosphate and distilled water. Subsequently, dehydration was performed in seven stages: 30% ethanol for 10 min, 50% ethanol for 10 min, 70% ethanol for 10 min, 90% ethanol for 10 min, 90% ethanol for 20 min, 100% ethanol for 10 min, and 100% ethanol for 20 min.

The samples were fixed on a support with double-sided carbon tape and metallized with a gold film (Quorum Q150R ES) at the Department of Mechanical Engineering of Instituto Tecnológico de Aeronáutica (ITA). Micrographs were obtained using SEM-FEG equipment (Tescan Mira 3) at the Coordination of Research and Technological Development of the Instituto Nacional de Pesquisas Espaciais (COPDT/INPE).

## 3. Results

### 3.1. O_3_ Concentration Curve Dissolved in Water

**Figure 2:**
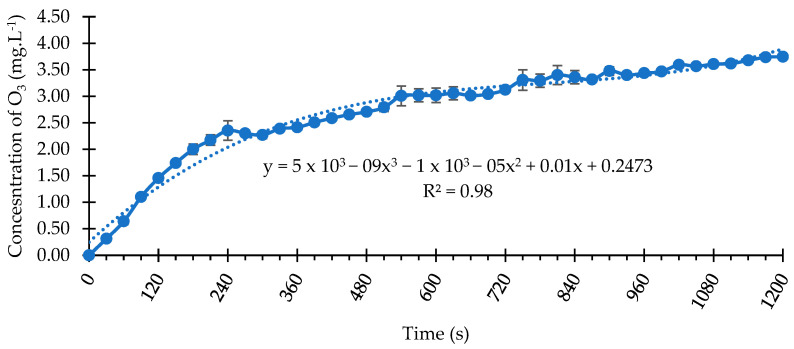
O_3_ concentration curve dissolved in water at 18 °C.

Figure 2 presents the O_3_ concentration curve with up to 1200 s (20 min) of prior ozonation. However, in this study, a time determined in a study carried out by Heinzelmann et al. [32], 600 s (10 min), was used, resulting in a concentration level of 3.0 mg/L. In the concentration curve analysis, the coefficient of determination was R^2^ = 0.98, indicating a high correlation between time and O_3_ concentration.

### 3.2. FT-IR Analysis

**Figure 3:**
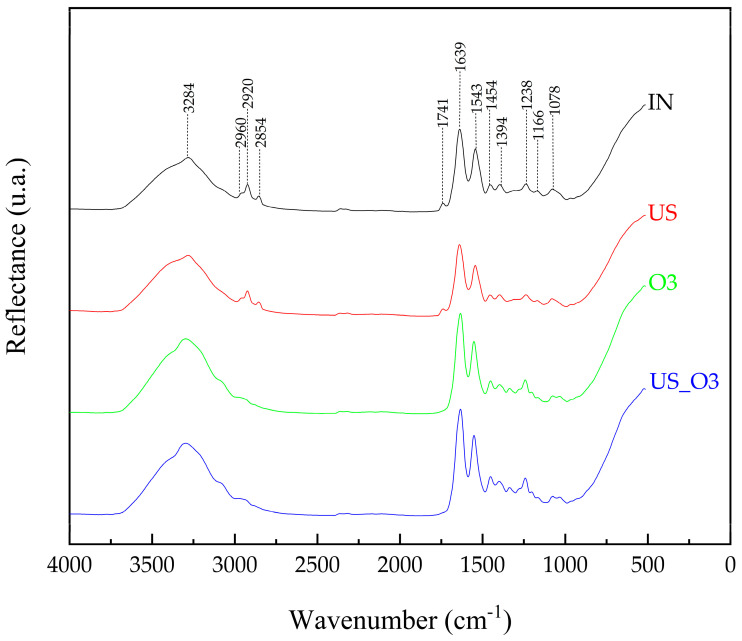
FT-IR spectra obtained from the analyzed samples: IN—Control/natural (black). US—Processed with ultrasound in liquid medium (red). O3—Processed with ozonated water (green). US_O3—Processed with ozonated water combined with ultrasound (blue).

The experimental FT-IR spectra and the assignment of the observed bands are presented in Figure 3. The spectra exhibited similarity; however, subtle changes can be highlighted, such as slight band shifts and intensity changes representing the biochemical characterization of the samples. Figure 3 presents the assigned values from the IN sample, which were then subsequently compared with the other samples. Table 1 presents the assignments of the functional groups and main bands in the HAM spectrum.

Amide A is characterized by NH stretching at 3300–3310 cm^−1^ [35]. In this study, a broad band was observed at approximately 3284 cm^−1^, with higher intensity in the O3 and US_O3 samples, while the US sample showed similarity to the IN sample.

The peak at 2960 cm^−1^ is attributed to the asymmetric stretching of CH_3_ [36], and the peak at 2920 cm^−1^ corresponds to the asymmetric stretching of CH_2_. Both characterize the presence of lipids, DNA, proteins, carbohydrates, and nucleic acids, while the band at 2854 cm^−1^, which is attributed to the symmetric stretching of CH_2_, only characterizes lipids [37]. In this study, when comparing the IN and US samples, a similarity was noted in both the O3 and US_O3 samples, and after processing in the system, the band intensity decreased, as did the band at 1741 cm^−1^, which is attributed to C=O stretching.

Amide I at 1639 cm^−1^ is attributed to C=O stretching [37,38]. In the samples, the bands remained the same. In the spectrum, amide II can be identified in the band at 1543 cm^−1^ [37], and it is attributed to NH bending and CN stretching modes [36,38]. This peak remained present in all the samples with intensities like that of the IN sample, remaining almost unchanged. Similarly, the band at 1394 cm^−1^, which is characteristic of the functional group of proteins attributed to the CH_3_ wagging mode [37], remained.

Amide III, identified in the spectrum at 1238 cm^−1^ [35], is characterized by CN stretching and NH bending modes [39], in addition to the contributions of CC stretching and CO bending [36]. In this study, the intensities of the US and IN samples remained similar, while in the O3 and US_O3 samples, an increase in peak intensity was observed. In the spectrum at approximately 1166 cm^−1^, the presence of a collagen (type I) functional group is observed, attributed to CO stretching at 1078 cm^−1^, the band corresponding to nucleic acids, glycolipids, and phospholipids, and at 1078 cm^−1^, related to PO_2_ stretching [37]. Compared with the IN sample, the other samples did not undergo changes and remained stable after the experiment.

### 3.3. Histological Analysis

**Figure 4:**
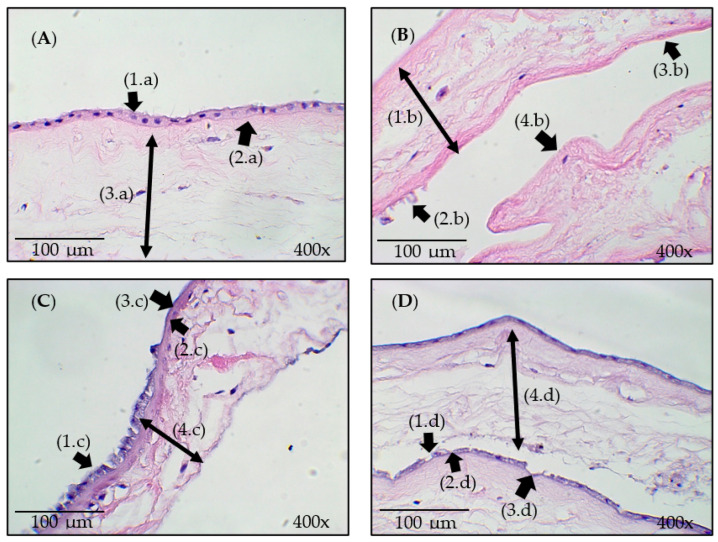
Histological images of the samples: (**A**) IN—Control/natural. (**B**) US—Processed with ultrasound in a liquid medium. (**C**) O3—Processed with ozonated water. (**D**) US_O3—Processed with ozonated water combined with ultrasound. The arrows (1.a), (2.b), (1.c), and (1.d) indicate epithelial cells. The arrows (2.a), (3.b), (2.c), and (2.d) indicate the basement membrane. The arrows (4.d), (3.c), and (3.d) indicate areas with epithelial cell removal. The arrows (3.a), (1.b), (4.c), and (4.d) indicate the stroma.

The histological images of the samples are presented in Figure 4. The structural changes that occurred in the HAM after processing in the system developed with ozonated water and ultrasound can be identified, as indicated by the arrows in this figure.

In Figure 4A, displaying the IN sample, the presence of intact cell junctions and cuboidal epithelial cells adhered to each other is indicated by the arrow (1.a). The intact basement membrane is indicated by the arrow (2.a), and the stroma with an organized collagen arrangement is indicated by the arrow (3.a).

The analysis of the histological image of the US sample presented in Figure 4B, as indicated by the arrow (1.b), shows that the stroma remained dense, with an appearance similar to that of the IN sample. The epithelial cells were almost completely removed, and a large area of tissue de-epithelization was noted, as indicated by the arrow (4.b). The remaining cells underwent significant changes, as indicated by the arrow (2.b), and areas of the basement membrane demonstrated small changes, as indicated by the arrow (3.b). Thus, it is possible that the mechanical effect of ultrasound and water flow alters the structural layers of the HAM epithelium, favoring de-epithelization.

In the O3 sample represented in Figure 4C, the absence and destruction of some epithelial cells are noted, as indicated by the arrow (1.c). Despite the alterations in the epithelial tissue, the basement membrane demonstrated small changes, as indicated by the arrow (2.c). The changes observed in the O3 sample show the removal of the epithelium in some regions of the HAM (3.c). As indicated by the arrow (4.c), the stroma showed a slight alteration in the arrangement of collagen fibers.

As shown in Figure 4D, slight alterations in the epithelial layer of the US_O3 sample were observed. In a few areas, the epithelial cells underwent structural deformation, as indicated by the arrow (1.d), and the basement membrane, indicated by arrow (2.d) remained preserved. Among the samples, the smallest area of epithelial cell removal was observed (3.d). The collagen fibers in the stroma appeared more dispersed, as shown by the arrow (4.d); however, their fundamental structure was not altered.

### 3.4. SEM-FEG Analysis

**Figure 5:**
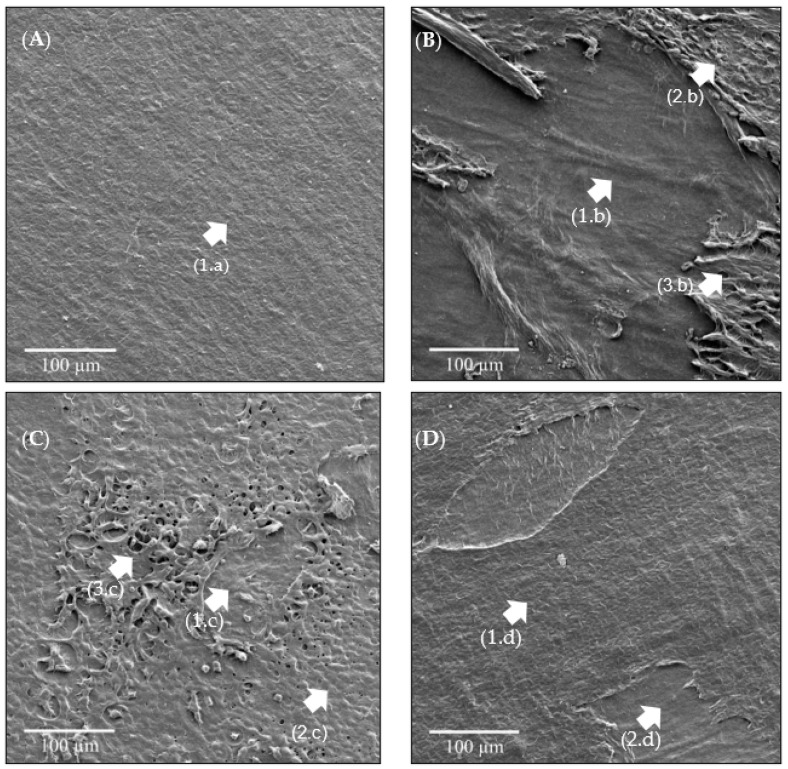
SEM-FEG images of the samples: (**A**) IN—Control/natural. (**B**) US—Processed with ultrasound in a liquid medium. (**C**) O3—Processed with ozonated water. (**D**) US_O—Processed with ozonated water combined with ultrasound. Arrows (1.a), (2.c), and (1.d) show intact epithelial cells. Arrows (2.b), (3.b), and (3.c) show irregular epithelial cells. Arrows (1.b), (1.c), and (2.d) show regions with epithelial cell removal.

The images of the samples obtained via SEM-FEG are presented in Figure 5. The visualized structures corroborate those identified in Figure 4. In Figure 5A, the IN sample is visualized with its epithelial layer clearly defined by the sharpness of uniformly adhered polygonal epithelial cells, characterizing a regular surface pattern with clearly defined intercellular spaces, as indicated by the arrow (1.a).

In Figure 5B, presenting the US sample, the removal of the epithelium in a large area of the HAM is noted (1.b), with few regions containing epithelial cells characterized by an irregular surface, as indicated by the arrows (2.b) and (3.b). In the O3 sample shown in Figure 5C, in some regions, the cuboidal epithelium was removed (1.c), while in others, it remained intact (2.c). An area of the HAM surface showed roughness and overlapping of epithelial cells (3.c). The analysis of the US_O3 sample shown in Figure 5D revealed that the epithelial tissue underwent morphological alterations; however, in a large area, the epithelial cells remained intact (1.d), and areas with removed epithelial tissue (2.d) remained intact.

**Figure 6:**
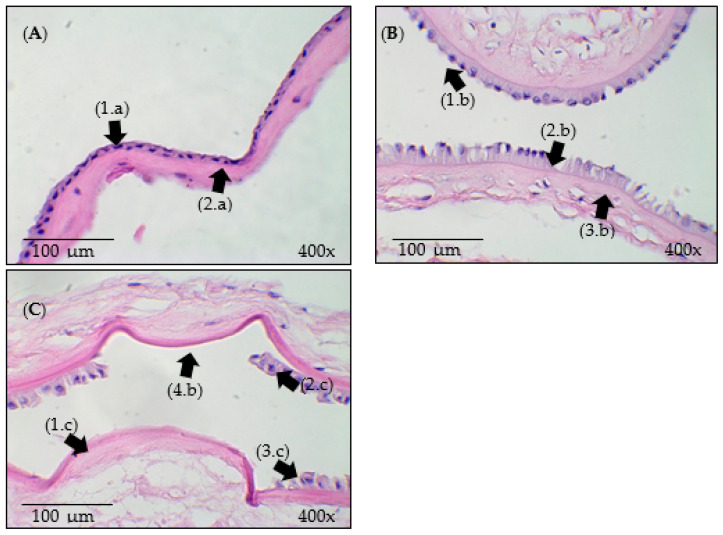
Histological images of the samples: (**A**) IN—Control/natural. (**B**) O2_US—Processed with water, oxygen, and ultrasound. (**C**) US—Processed with ultrasound in a liquid medium. Arrows (1.a), (1.b), and (3.c) indicate epithelial cells. Arrows (2.a), (3.b), and (4.c) indicate the basement membrane. Arrows (2.b), (1.c), and (2.c) indicate areas with epithelial cell removal.

As noted in the results, the US sample is more promising for the de-epithelization of the HAM. Since the US_O3 sample did not achieve the expected result, it was hypothesized that the bubbles generated by O_3_ influenced the cavitation effect of ultrasound. To test this hypothesis, three more samples were prepared and divided into the following groups: control/natural (IN_C), processed with O_2_ and ultrasound (O2_US), and processed with ultrasound in a liquid medium (US_C). These were subjected to the system for 55 min. Upon completion of the experiments, histological analysis was performed, as shown in Figure 6.

In Figure 6A, presenting the IN sample, as indicated by the arrow (1.a), cuboidal epithelial cells adhered to each other, and the presence of the basement membrane is indicated by the arrow (a.2). According to the histological image of the O2_US sample shown in Figure 6B, areas demonstrating epithelial tissue integrity are indicated by the arrow (1.b), areas demonstrating structural alterations at the cell edges are indicated by the arrow (2.b), and the arrow (3.b) indicates the intact basement membrane.

When viewing the image of the US sample, the arrow (1.c) shows areas that underwent complete de-epithelization, as well as a homogeneous detachment of the epithelial tissue layer indicated by the arrow (2.c) with the presence of some cells, all with structural alterations, as well as in the basement membrane, indicated by the arrows (3.c) and (4.c), respectively.

Therefore, the results found are similar to those noted in the initial experiment. It can be concluded that the bubbles generated by the system, whether O_2_ or O_3_, alter the vibrational effects of ultrasound cavitation and interfere with the process of epithelial cell removal from the HAM.

## 4. Discussion

In this study, the biochemical composition and de-epithelization of the HAM after the use of a system involving ozonated water and ultrasound were verified. It was noted that when only using an ultrasound system, the effect of de-epithelization resulted in small structural changes in the basement membrane. Traditional de-epithelization methods, however, cause alterations in the HAM stroma [40] and spectral differences [31].

The use of ultrasound in the de-epithelization of the HAM involves the use of a low-frequency (24–40 kHz) ultrasonic bath combined with glycerol and/or lyophilization. In these methods, spectral differences occur between them [31]; however, the spectra obtained in this study showed similarities, mainly when comparing the IN and US samples. The functional group of amides A, I, II, and III were identified [41].

In tissue engineering, the extracellular matrix is of fundamental importance in the outcome of clinical responses [42]. A study conducted by Cavalu et al. [36] evaluating membrane processing with gentamicin and ultraviolet exposure showed that the removal of epithelial cells resulted in collagen fiber division.

The de-epithelization of the HAM with trypsin, EDTA, and thermolysin does not completely remove epithelial cells, and dispase leads to stroma fragmentation [43], which was also observed in the histology and SEM-FEG results of this study concerning the O3 and US_O3 samples, where epithelial cells and structural tissue deformation were noted. Notably, compared with the other samples, the US sample had the lowest number of epithelial cells and a denser stroma. The other samples showed alterations in the collagen fiber arrangement of the stroma.

Awoyama et al. [18] used a system developed with ozonated water and noted small morphological changes in the HAM epithelial layer. In this sense, the results of the O3 sample were like those of this study, showing that ozonated water modifies the shape and removes small areas of the epithelial layer with small changes to the basement membrane.

A recent study using ozonization revealed that epithelial cells showed structural alterations, and the longer the exposure time was, the thinner the average epithelium was [44]. In this study, structural alterations in the cell surface were observed, making the cell surface irregular and undefined in shape.

No study that uses the hydrodynamic action of water and ultrasound has been reported in the literature. A study using a low-frequency ultrasonic bath revealed the removal of the epithelium in large areas of the membrane, but significant damage to the epithelial layer also occurred, with basement membrane desquamation, stroma exposure, and trabecular architecture formation [31].

Unlike the previously mentioned study, this study used a system with an average water speed of 2 cm/s, which, when combined with ultrasound cavitation, may have potentiated the effects of de-epithelization, stroma preservation, and the biochemical properties of the HAM.

Fewer alterations in the epithelium were observed in the US_O3 sample than in the O3 and US samples. This can be attributed to the bubbles present in the liquid medium, which may interfere with vibrational effects and ultrasound cavitation.

Water, bubbles, and the HAM have different densities, and the ultrasonic wave initially reflects off the bubbles due to their lower density. As a result, both the bubbles and the ultrasonic waves reduce their interaction with the HAM. Because there is a difference in acoustic impedance between the materials, this difference leads to either the greater or lesser reflection of the sound waves [45].

## 5. Conclusions

After HAM was processed in the system, the following conclusions were drawn:

- The FT-IR spectra showed subtle changes, such as slight shifts and intensity changes in the bands, indicating no significant changes in the biochemical properties of the HAM after the experiment.
- Ultrasound had greater effects on de-epithelization, and O_3_, despite causing similar effects, did not surpass the impact of ultrasound.
- In the case of ozonated water combined with ultrasound, no significant alterations in the structure of the epithelial tissue were observed.
- Therefore, cavitation caused by the ultrasound promoted the de-epithelization of the HAM, without significant changes to the stroma.

These findings indicate that this system has great potential in the processing of HAM. It is more advantageous than traditional methods in preserving the stroma, speeding up de-epithelialization, and has no environmental impact.

It is recommended that new studies be carried out using this system to evaluate other possible uses. From this, it will be possible to verify its advantages in terms of disinfection and sterilization when using ozonated water and de-epithelialization with ultrasound, defining the best, most complete HAM preparation protocol. 

This study has limitations related to the use of fresh biological material, which presented challenges in terms of obtaining samples.

## Figures and Tables

**Table 1:** Assignment of functional groups and main bands of the HAM spectrum.

Functional Group	ω (cm^−1^)	Assignments	Reference
Amide A	3284	ν NH	[35,36]
Lipids, DNA and proteins, carbohydrates and nucleic acids	2960	ν_a_ CH_3_	[36,37]
	2960	ν_a_ CH_3_	[36,37]
Lipids	2854	ν_s_ CH_2_	[36]
Fat and lipid	1741	ν C=O	[36]
Amide I	1639	ν C=O	[36,38]
Amide II	1543	sc NH/ν C-N	[36,37,38]
Lipids	1454	sc CH_2_	[36]
Proteins	1394	wag CH_3_	[36]
Amide III	1238	ν C-N/sc N-H/ν C-C/sc C=O	[35,37,39]
Collagen (type I)	1166	ν C-O	[36]
Nucleic acids, glycolipids, and phospholipids	1078	ν PO_2_	[36]

ω—Wavenumber. ν—Stretching. νa—Asymmetric stretching. νs—Symmetric stretching. sc—Scissoring (bending). wag—Wagging.

## Data Availability

Data are contained within the article.
